# Supplementary material for: Content-rich biological network constructed by mining PubMed abstracts
Source: BMC Bioinformatics. 2004 Oct 8;5:147. doi: 10.1186/1471-2105-5-147 (PMC528731; doi:10.1186/1471-2105-5-147)
Supplement: Additional File 5 — The original Chilibot query results of the term "long-term potentiation (LTP)" and 22 other terms, limiting the latest references analyzed to the years 1990, 1995, 2000, and 2004. [file 1471-2105-5-147-S5.bz2 › chilibotAdditionalFile5/ltp2004/html/ACTIN_SYNAPTOTAGMIN.html]

 


 **ACTIN** and **SYNAPTOTAGMIN** 
  
Found 13 abstracts in PubMed,  **13 abstracts were retrieved and analyzed**.  


---

 Search Google  |
 PDF files only 
|  EDU domain only 

---

**Interactive relationship** (e.g. stimulation, inhibition, etc)

- Slac2 c  **synaptotagmin**  like protein homologue lacking C2 domains c, a novel linker protein that interacts with Rab27, myosin Va VIIa, and  **actin** .  Ref: 12221080 J Biol Chem, 2002
- Slac2 a  **synaptotagmin**  like protein Slp homologue lacking C2 domains a melanophilin is a melanosome associated protein that links Rab27A on melanosomes with myosin Va, an  **actin**  based motor protein, and formation of the tripartite protein complex Rab27A.Slac2 a.myosin Va has been suggested to regulate melanosome transport Fukuda, M.  Ref: 12221080 J Biol Chem, 2002

**Parallel relationship** (e.g. studied together, co-existance, homology, etc.)

- Here we show that fibroblast cell lines transfected with  **synaptotagmin**  form long, highly branched,  **actin**  rich filopodial processes, with the expressed  **synaptotagmin**  being incorporated into the plasma membrane.  Ref: 8166886 Nature, 1993
- In PC12 cells, Nadrin colocalized with  **synaptotagmin**  in the neurite termini and also with cortical  **actin**  filaments in the subplasmalemmal regions.  Ref: 10967100 J Biol Chem, 2000
